# Regional probabilistic risk assessment of heavy metals in different environmental media and land uses: An urbanization-affected drinking water supply area

**DOI:** 10.1038/srep37084

**Published:** 2016-11-15

**Authors:** Chi Peng, Yimin Cai, Tieyu Wang, Rongbo Xiao, Weiping Chen

**Affiliations:** 1 State Key Laboratory of Urban and Regional Ecology, Research Center for Eco-environmental Sciences, Chinese Academy of Sciences, Beijing, 100085, People’s Republic of China; 2 Guangdong Provincial Academy of Environmental Science, Guangdong, 510045, China

## Abstract

In this study, we proposed a **R**egional **P**robabilistic **R**isk **A**ssessment (RPRA) to estimate the health risks of exposing residents to heavy metals in different environmental media and land uses. The mean and ranges of heavy metal concentrations were measured in water, sediments, soil profiles and surface soils under four land uses along the Shunde Waterway, a drinking water supply area in China. Hazard quotients (HQs) were estimated for various exposure routes and heavy metal species. Riverbank vegetable plots and private vegetable plots had 95^th^ percentiles of total HQs greater than 3 and 1, respectively, indicating high risks of cultivation on the flooded riverbank. Vegetable uptake and leaching to groundwater were the two transfer routes of soil metals causing high health risks. Exposure risks during outdoor recreation, farming and swimming along the Shunde Waterway are theoretically safe. Arsenic and cadmium were identified as the priority pollutants that contribute the most risk among the heavy metals. Sensitivity analysis showed that the exposure route, variations in exposure parameters, mobility of heavy metals in soil, and metal concentrations all influenced the risk estimates.

Rapid urbanization and industrialization have released toxic chemicals such as heavy metals into the environment, threatening human health^1^,^2^. Pollution assessment methods such as the Pollution Index and Enrichment Factors are commonly used to rapidly evaluate pollution levels in specific sampling sites that adopt national environment standard values or natural background values as the reference concentrations^3^,^4^. These assessments ignore the actual connections between contaminants and human health and are inherently inadequate to address how human behaviors and land use patterns affect the health risks posed by pollution. Health risk assessments evaluate the probability of deleterious effects by constructing conceptual risk models in terms of risk sources (pollutants in various environmental media), exposure routes, and risk receptors (the sensitive population)^5^,^6^. Health risk assessment was first used in field-scale studies such as evaluating contamination at Superfund sites^6^. Health risk assessments are therefore capable of distinguishing contaminated sites from virtually safe areas, identifying pathways of the highest risks for a given land use or at a site, and guiding the control of priority pollutants that are threatening human health^7^,^8^,^9^.

Health risk assessment retains large uncertainties in the procedure of exposure assessment^10^. Using soil as an example, the exposure routes of heavy metals in the environment include oral intake, dermal contact and inhalation. Calculating exposure frequency and durations of heavy metals to the sensitive population involves many site- or chemical-specific parameters^11^. Traditional health risk assessment is a deterministic process that adopts the reasonable maximum exposure parameters to obtain a conservative result^12^. However, deterministic risk assessments may under- or over-estimate the risks and, hence, are unconvincing^13^. In contrast, probabilistic risk assessment (PRA) attempts to characterize uncertainty and variability by calculating the risks based on the range and statistical distribution of the exposure parameters^14^. Thus, PRA provides complete and detailed risk information for environmental management^15^. Practical illustrations of PRA were limited because the computation processes and input data collection are more complicated and difficult than other assessment techniques^16^. Nevertheless, PRA is necessary for obtaining the risk distributions and evaluating the importance of each exposure route or input parameter in affecting the total risks.

Recently, regional heavy metals pollution caused by urbanization and industrialization has drawn great attention^2^,^17^. Different environmental media and land uses dramatically alter heavy metals exposure routes and the sensitive population. For example, river sediment may threaten only the population that swims periodically, whereas residential soils pose potential risks to children playing on the ground, and agricultural soils are sources of exposure to farmers^5^,^18^,^19^. The consideration of multiple media and multiple land uses in risk assessment introduces more parameters and uncertainties into the analysis^16^. Therefore, the traditional health risk assessments commonly focused on single media or land use^12^,^13^,^20^,^21^. At a regional scale, PRA would be more suitable for evaluating health risks than deterministic risk assessment as it can provides statistical information that explains spatial variations and individual differences^20^. It is imperative to include multimedia and land uses into regional-scale PRA. An ideal way is to identify firstly risk receptors (the most sensitive population) and exposure routes according to each media and land use, and then calculate risk probabilities respectively.

Drinking water supply areas in China are facing challenges because rapid urbanization has extended the sphere of urban influence^17^. Previous research showed that heavy metals accumulating in vegetable soils of flooded riverbank were significantly higher than non-flooded vegetable soils^11^. The Shunde Waterway, which is the drinking water source for the cities of Foshan and Guangzhou, was once located far away from the cities but has become incorporated into the suburban areas of Foshan during the urban sprawl process (Fig. S1). The increasing activity of the manufacturing industry in this area is threatening the safety of drinking water sources, and the growth of population has caused conversion of once-barren land to public green space and vegetable plots. The levels of risk exposure to heavy metals in this area are of concern, especially in vegetable soils near the flooded riverbank. In the study described herein, **R**egional-scale **P**robabilistic **R**isk **A**ssessment (RPRA) was proposed to evaluate the health risks of heavy metals in the Shunde Waterway area as a function of different media and land uses. The purposes of the study were to demonstrate the application of PRA on regional scale and to identify heavy metal species that pose the highest risks and the priority exposure routes.

## Materials and Methods

### Procedures of probabilistic risk assessment at regional scale

**Figure 1:**
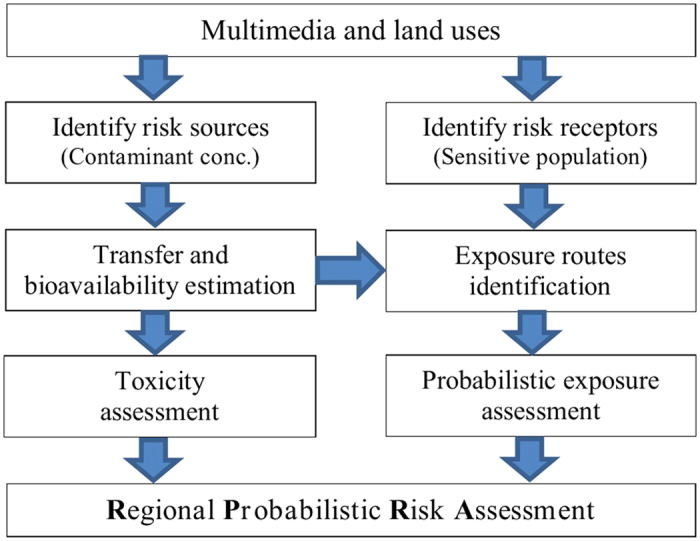
Framework of Regional-scale Probabilistic Risk Assessment (RPRA).

Health risk assessment involves several general procedures including (1) identification of risk sources and receptors, (2) exposure assessment, (3) toxicity analysis and (4) risk characterization^6^. Based on the general procedures of health risk assessment, we proposed a framework for regional probabilistic risk assessment under different land use and environmental media (Fig. 1). The risk sources are the heavy metals in the various environmental media, and the risk receptors are the most sensitive population varying with the media and land use types. The exposure routes and frequencies vary according to daily behaviors of the sensitive population. Coupled with Monte Carlo simulation, RPRA incorporates parameter variation into the calculation of the first two steps. The individual differences and spatial variations are represented by the ranges and distributions of the data parameters.

Non-carcinogenic risks arising from exposure to heavy metals in three media (water, sediment and soil) and two land uses (green space soil and agricultural soil) were evaluated. For soil, the sensitive population of green space was considered to be the residents who have regular outdoor activities; and the sensitive population of vegetable plots was farmers who worked on the sites. Exposure routes included incidental oral ingestion of soil particles, dermal contact with soil particles and inhalation of soil dust during outdoor activities. Additional risks posed by ingestion of groundwater and vegetables, by which heavy metals were transferred from soils, also were calculated. For water and sediments, the sensitive population was comprised of residents who have regular swimming activities. The exposure routes for these residents included the daily intake of water, incidental intake of sediment and dermal contact with water and sediment during swimming. The carcinogenic risks were not considered in the current study due to most of the metals are lack of validated carcinogenic slope factor to evaluate their carcinogenic effect^8^.

### Risk assessment models

The non-carcinogenic health risks represented as hazard quotients (HQs) are equal to the chronic daily intake (*CDI*) divided by the chronic reference dose (*RfD*). Therefore, the risk assessment models vary depending on exposure routes such as oral ingestion, dermal contact and inhalation.

For ingestion of soil particles, sediment particles, surface water and ground water, the health risk models can be written as Eq. (1)^7^,^22^:


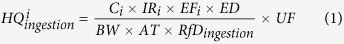


where *i* represents a target media or land use. *IR*_*i*_ represents the ingestion rate, including soil ingestion rate *IR*_*s*_ (mg/d), sediment ingestion rate *IR*_*sd*_ (mg/d), daily water ingestion rate *IR*_*w*_ (mL/d); and *EF*_*i*_ represents the exposure frequency (d/yr) for indoor activities *EF*_*ia*_, outdoor activities *EF*_*oa*_ and swimming *EF*_*sd*_, respectively. *ED* indicates the exposure duration (yr), *BW* indicates the average body weight (kg), and *AT* indicates the average total time (d). *RfD*_*ingestion*_ denotes the chronic oral reference dose (mg/kg/d). *UF* represents a unit transfer factor, which is ×10^−6^ for soil and sediment and ×10^−3^ for water. *C*_*i*_ indicates the metal concentrations in the target media or land use (mg/kg for soil and sediment and μg/L for water).

The metal concentrations in groundwater *C*_*gw*_ (μg/L) were calculated using a leaching equation and a dilution factor (*DF*) (Eq. 2)^7^:


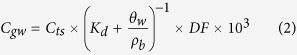


where *C*_*ts*_ (mg/kg) indicates the metal concentrations in the total soil profile (0–100 cm), *K*_*d*_ represents the soil-water partition coefficient (L/kg), *θ*_*w*_ represents the water-filled soil porosity (L_water_/L_soil_) and *ρ*_*b*_ is the dry soil bulk density (g/cm^3^).

The risks arising from intake of heavy metals from vegetables planted in soil can be written as Eq. (3)^14^:


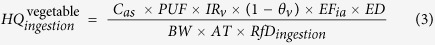


where *C*_*as*_ (mg/kg) indicates the metal concentrations in the agricultural soils (0–20 cm), *PUF* (unit-less) represents the plant uptake factor, *IR*_*v*_ indicates the vegetable ingestion rate (kg/d), and *θ*_*v*_ (unit-less) is the vegetable water content.

For dermal contact with soil, sediment and water, the health risk model is written as Eqs (4) and (5)^7^,^22^:

For soil and sediment


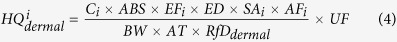


For water


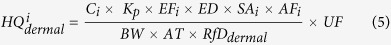


where *ABS* (unit-less) represents the dermal absorption factor for soil and sediment. *K*_*p*_ (cm/h) represents the dermal permeability constant for heavy metals in water. *SA*_*i*_ (m^2^) represents the skin surface area available for exposure in outdoor activities or in swimming. *AF*_*i*_ (mg/cm^2^) represents the soil-to-skin adherence factor for farmers and adults. *RfD*_*dermal*_ (mg/kg/d) is the chronic reference dose through dermal contact and *UF* represents a unit transfer factor (×10^−6^).

For inhalation routes, the health risk model for risks posed by inhalation of soil particles and vapors is Eq. (6)^7^,^22^.


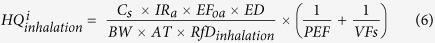


where, *IR*_*a*_ (m^3^/d) is the air inhalation rate, *RfD*_*inhalation*_ (mg/kg/d) is the chronic inhalation reference dose, *PEF* (m^3^/kg) indicates the particulate emission factor and *VF* is the volatilization factor, which in this study was relevant only for elemental Hg (32,376.4 m^3^/kg)^10^.

The total hazard quotient (*THQ*) is the sum of individual *HQs* for each media or land use, *i,* and is given by Eq. (7):


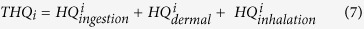


in which 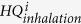 is calculated only for heavy metals in surface soils. Values of HQ and THQ greater than 1 indicate potential health risks while those less than 1 suggest acceptable risks.

### Models parameters

Parameters for exposure frequency and duration, metal concentrations, and reference toxic dose are necessary to apply RPRA. The mean and ranges of the exposure parameters in the study area were collected mostly from the Chinese Exposure Factors Handbook^23^, in which nearly 100,000 questionnaires and surveys in China were compiled to obtain site-specific exposure parameters, such as the frequency of swimming, ingestion rate of water, air and vegetables, skin surface area and average body weight. The frequencies of indoor and outdoor activities were set to 350 d/yr and 225 d/yr, respectively^23^,^24^. The mean and ranges of *PUF*, *θ*_*v*_, *θ*_*v*_, *ρ*_*b*_*, DF*, *IR*_*sd*_ and *AF*_*sd*_ were taken from published sources^14^,^19^,^25^,^26^,^27^. Other exposure parameters and chronic reference toxic doses (*RfD*) were taken from USEPA documents^6^,^22^,^24^. The values and distributions of the model parameters used in the current study are summarized in the Supplementary information (Tables S1 and S2). Parameters on heavy metal concentrations and soil *pH* were measured in field investigations.

### Field data collection

Fifty-one surface soil samples (0–20 cm), 6 soil profiles (0–100 cm), 10 surface water samples and 10 river sediment samples were collected along the Shunde Waterway to obtain the mean and ranges of heavy metal concentrations in the study area for different media and land uses. The distribution of sampling sites is shown in Fig. S1. The sampling sites of surface soils can be classified into four categories according to their land use types, including riverbank vegetable plots (RVS, n = 7), riverbank green spaces (RGS, n = 13), private vegetable plots (PVS, n = 11) and public green spaces (PGS, n = 20). The RVS and RGS sites are close to the river while PVS and PGS sites are located further away from the riverbank, but still within 200 m to 2000 m from the river. Each surface soil sample was the composite of five subsamples collected within a 100 m^2^ area at each sampling site. The soil profiles were randomly sampled from vegetable plots, two of which were located at the riverbank and four were away from the riverbank. Each soil profile sample was the composite of five subsamples, each taken at 20-cm increments from the 100-cm deep profile. Water and sediment samples were randomly collected at 10 riverbank sites (near to the RVS and RGS sites). The concentrations of As, Hg, Cd, Zn, Pb, Cu, Cr and Ni in the samples were analyzed by inductively coupled plasma-mass spectroscopy and inductively coupled plasma-optical emission spectrometry after acid digestion^28^.

### Monte Carlo simulation

Monte Carlo simulations were conducted using Crystal Ball 11.1. Denver, USA. For each exposure parameter or metal concentration, the trial values were generated according to their mean value and data range. The statistical distributions for the parameters were primarily set to lognormal distributions, but some others were triangular distributions (Table S1). The dependences of parameters, including the correlations between *BW* and *SA* as well as between *PUF* and *K*_*d*_, were assumed during the generation of trial values. To obtain robust results, each Monte Carlo simulation was set to 10,000 iterations for each of the parameters. Then the health risks were calculated by using the generated 10,000 parallel groups of the model parameters in Equations (1, 2, 3, 4, 5, 6).

## Results and Discussion

### Heavy metal concentrations

The concentrations of heavy metals in the water, sediments and soils from different land uses along the Shunde Waterway are summarized in Table 1. The concentrations of As, Cd, Zn, Pb, and Cu in the riverbank soils of RVS and RGS were significantly higher than those in the soils of PVS and PGS, respectively, and were higher than background values (Table 1) suggesting external inputs of these metals from anthropogenic activities. The sediments had concentrations of the heavy metals that were similar to the riverbank soils (Table 1). The riverbank soils receive deposits of suspended particulates from the river; these deposits provide nutrition for the growth of vegetables and ornamental vegetation. The anthropogenic As, Cd, Zn, Pb and Cu in the riverbank soils were most likely released from industrial facilities or villages in the upstream portion of the watershed and then transported downstream by water flow before being deposited on the riverbank. Rapid urbanization has caused the prosperity of the manufacturing industry in Shunde County, such as plastic materials production, furniture production, small appliances manufacturing, and textile and garment production^29^. The scattered and small manufacturing plants without strict supervision may releases heavy metals into the water network.

On the contrary, the soils of PVS and PGS, which were located away from the river, received heavy metals mainly from atmospheric deposition. These soils have slightly higher concentrations of As, Hg, Cd and Zn than the background values (Table 1). Cai, Xu^30^ reported that the Cd and As found in Shunde County were mainly related to industrial and agronomic practices, while Hg was released from coal burning, industrial fumes and traffic emissions. Atmospheric deposition is the major input pathway of Hg in the soils and leads to the small differences in Hg concentrations between the riverbank soils and other soils. The concentrations of Pb, Cu and Ni in the soils of PVS and PGS were slightly lower than the reported background values (Table 1). Because the soil backgrounds of heavy metals are spatially varied and their measurements can be affected by the choice of sampling sites and analytical methods^31^.

Compared with previous studies, the soil concentrations of As, Hg, Cd, Zn and Pb in the riverbank of the Shunde Waterway are considerably higher than that in the water source protection areas of Shanghai, Beijing, and the Pearl River delta^17^,^32^,^33^. The high concentrations of heavy metals in the riverbank soil and sediments along the waterway may threaten the health of local residents during outdoor recreation activities and consumption of vegetables planted on the riverbank. Therefore, identifying the risk levels of exposure to heavy metals in the study area is imperative.

### Risks of different media and land uses

Table 2 summarizes the mean and 95^th^ percentile values of HQs resulting from exposure to the heavy metals in different media and land uses. The 95^th^ percentiles of HQs were widely used to provide conservative and protective risk estimates^19^. The 95^th^ percentiles of *THQs* in the study area follows the gradient of RVS (3.57) > groundwater (1.46) > PVS (1.20) > surface water (0.84) > RGS (0.41) > river sediments (0.18) > PGS (0.17); the first three of these THQ values exceeds 1, suggesting potential health risks. The highest THQ was found in the RVS soils where the highest metal concentrations were measured. This finding suggests that the vegetable plots along the Shunde Waterway are threatening the health of local residents, especially farmers. In addition, the fertilization in riverbank vegetable plots may directly pollute the drinking water sources. Therefore, cultivation of edible crops on the riverbank soils should be discouraged.

THQs are mainly dependent on the exposure routes and exposure frequencies that vary with media and land uses. The vegetable plot soils (RVS and PVS) had significantly higher THQs than the green space soils (RGS and PGS) (Table 2). The hazard quotients for the exposure route of vegetable ingestion contributed most to the THQs of RVS and PVS. Therefore, even though the riverbank soils of RGS had relatively high metal concentrations, they had low THQ due to the absence of risk from vegetable ingestion.

**Figure 2:**
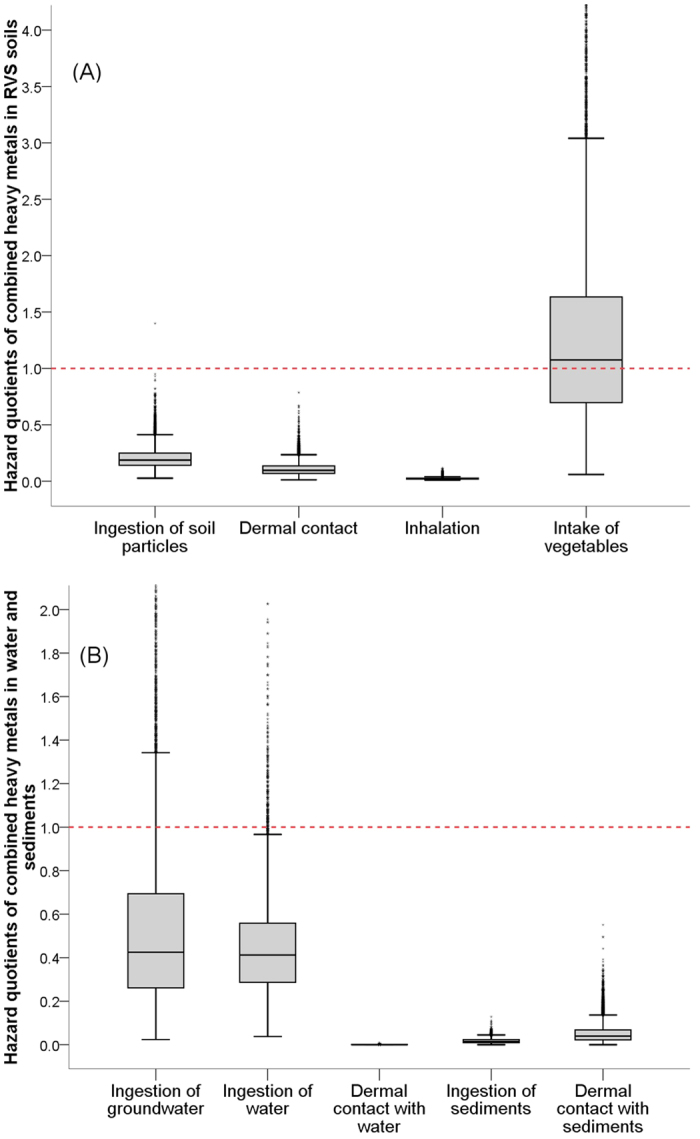
Box plots of hazard quotients for different exposure routes along the Shunde Waterway (The band inside the box represent the 50^th^ percentile and the whiskers indicates 1.5 IQR from the 25^th^ and 75th percentile).

**Figure 3:**
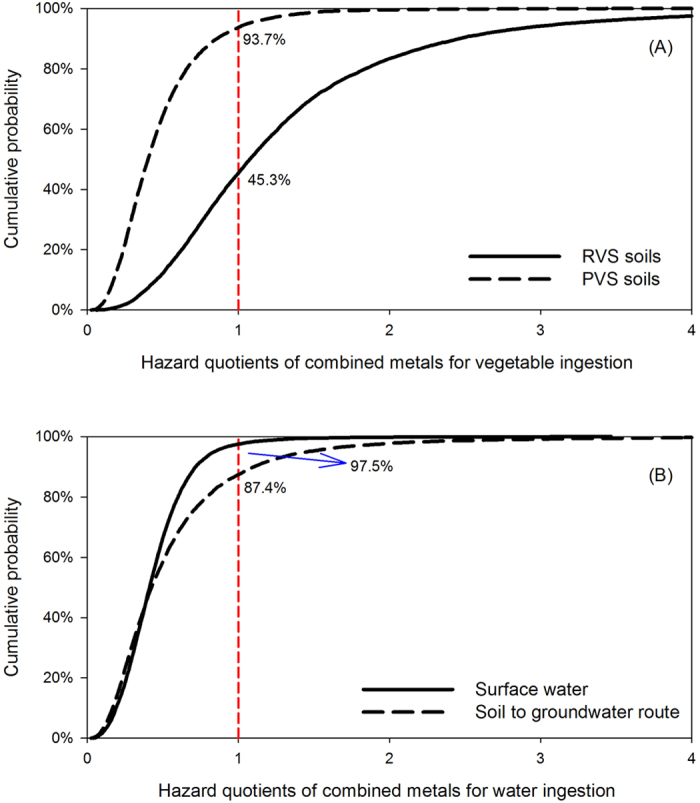
Cumulative probability of hazard quotients for vegetable ingestion and groundwater ingestion. (RVS = riverbank vegetable plot soils; PVS = private vegetable plot soils 200–2000 m from the riverbank).

Figure 2 presents the box plots of the HQs in terms of the exposure routes, from which the major causes of the risks can be identified, namely vegetable ingestion, groundwater ingestion and water ingestion. Considering the individual differences and spatial variations, the *HQ*_*ingestion*_ of vegetables in the RVS and PVS soils have 54.7% and 6.3% probabilities to exceed the threshold value of 1, respectively (Fig. 3A). The ingestion of groundwater and surface water have 12.6% and 2.5% chances, respectively, to exceed the safe level of heavy metal intake (Fig. 3B). The *HQ*_*ingestion*_ for groundwater was calculated based on the leaching potential of heavy metals in vegetable soils according to Eq. (2). The estimated heavy metal concentrations in the groundwater were close to the measured concentrations in surface water. Therefore, the mean values of *HQ*_*ingestion*_ between the groundwater and surface water were similar. Due to the large variances of *K*_*d*_ and *DF* (Supplementary information), the *HQ*_*ingestion*_ of groundwater had a considerably larger coefficient of variation and higher 95th percentile value than that of surface water (Table 2). Overall, metal uptake by vegetables and metal leaching to groundwater are the two important transfer routes of heavy metals in soils and may threaten human health. To reduce these health risks, measures should be taken such as converting vegetable fields to green space, or applying soil amendments that will immobilize heavy metals and prevent crop uptake.

Figure 2B illustrates that low HQs arise from ingestion of sediment and from dermal contact with water and sediment. These HQs are calculated based on the swimming frequencies of local residents. Neither *HQ*_*ingestion*_, 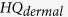
and *HQ*_*inhalation*_ in soil under the four land uses had a 95^th^ percentile value that exceeded 1 (Table 2). These results suggest that the outdoor activities such as recreation, farming and swimming along the Shunde Waterway are theoretically safe for human health.

### Risks of heavy metal species

**Figure 4:**
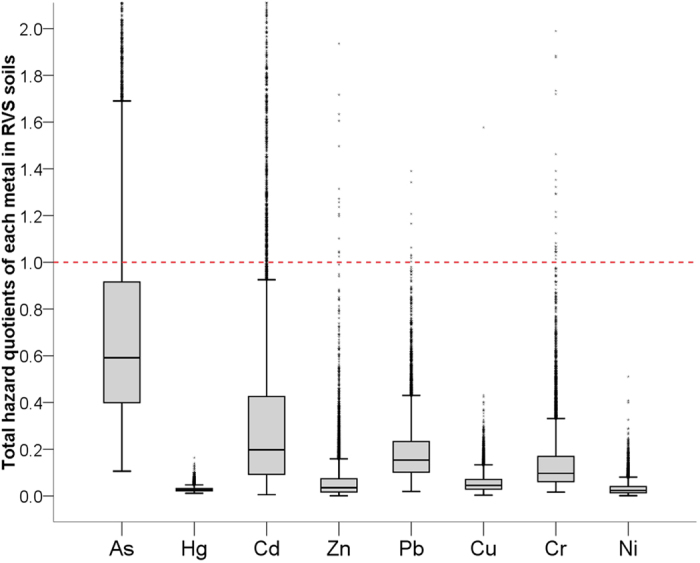
Box plots of total hazard quotients for metal species in RVS soils (riverbank vegetable plots).

Knowledge about the risk contributions of individual metal species to the total risks is essential for identifying the priority pollutants for remediation and emission control. The HQs varied dramatically with heavy metal species due to differences in toxicity, concentrations and mobility. Figure 4 shows that the 95^th^ percentile HQs follow the gradients of As (1.88) > Cd (1.30) > Pb (0.44) > Cr (0.39) > Zn (0.21) > Cu (0.13) > Ni (0.09) > Hg (0.05) in RVS soils. Similar risk gradients of the metal species were found in other land uses suggesting that As and Cd should be primarily considered for pollution management in the study area.

For different exposure routes, the eight heavy metals showed varying contributions to the HQs (Table 3). For instance, As contributed 80% and 92% of *HQ*_*ingestion*_ and *HQ*_*dermal*_ for RVS sites respectively, and 61% and 77% of that for PVS sites respectively. More than 98% of *HQ*_*inhalation*_ are caused by the volatilization of Hg; the contribution of other metal species is negligible. In RVS soils, As and Cd contributed 38.4% and 28.7% to *HQ*_*ingestion*_ of vegetables respectively, followed by Pb and Cr. Arsenic also contributed more than 80% of *HQ*_*ingestion*_ for groundwater and surface water. The results suggest that As and Cd were the primary risk sources in the high-risk exposure routes, including groundwater ingestion and vegetable ingestion. Therefore reducing As and Cd emissions or immobilizing them in the soils would be effective ways to control the health risk levels arising from these two contaminants.

### Sensitivity analysis

RPRA was used to estimate the probabilities of health risks based on the probabilities of input parameters. Consequently, the HQs showed large coefficients of variation ranging from 36% to 95% (Table 2). The variances of HQs were mainly dependent on the uncertainty and variability of the metal concentrations and exposure parameters. Correlation analysis was used to rank the importance of the input parameters on THQs for different media and land uses. Table 4 shows that *PUF*, *IR*_*v*_ and *θ*_*v*_ were the key factors affecting the health risks associated with vegetable plots, while As concentrations in soil, *IR*_*s*_ and *AF*_*sa*_ were important determinants of the health risks associated with green space. The risks of groundwater ingestion were highly related to soil As concentrations, *IR*_*w*_ and *DF*. Meanwhile, the *THQ*_*Water*_ was affected mostly by *IR*_*w*_ and by the As and Cr concentrations in the water. These results indicate that in estimating health risks, the variations of exposure parameters and changes of metal mobility in soil may be more important than the metal concentrations. Traditional health risk assessment that adopts default values for exposure parameters is challenged to reflect the authentic levels of risks. RPRA, which includes the uncertainty and variability of exposure parameters in the risk calculations, is a powerful technique by which to identify the exposure routes of high risks and the key factors affecting the risk levels.

## Conclusions

This research conducted regional-scale probabilistic risk assessment to estimate the health risks of exposure to heavy metals in water, sediments and soils for different land uses along the Shunde Waterway, a drinking water supply area in China. The risk receptors were set to the most sensitive population according to each media and land use. The Monte Carlo simulation was adopted to characterize the uncertainty and variability of exposure parameters and metal concentrations.

The 95^th^ percentile total hazard quotients for vegetable plots on the riverbank and further away (200–2,000 m) were 3.57 and 1.20, respectively, suggesting that these land uses pose potential threats to human health. Uptake by vegetables and leaching to groundwater are the two major transfer routes of heavy metals in soils that may threaten human health. The results indicated that cultivation on the flooded riverbank should be discouraged. Outdoor activities such as recreation, farming and swimming along the Shunde Waterway are theoretically safe. The metals As and Cd in soils are major risk sources and pollutants that should be given priority for management. The presence of high-risk exposure routes and variations in sensitive exposure parameters have more influence on health risk estimates than do heavy metal concentrations.

## Additional Information

**How to cite this article**: Peng, C. *et al.* Regional probabilistic risk assessment of heavy metals in different environmental media and land uses: An urbanization-affected drinking water supply area. *Sci. Rep.* **6**, 37084; doi: 10.1038/srep37084 (2016).

**Publisher’s note:** Springer Nature remains neutral with regard to jurisdictional claims in published maps and institutional affiliations.

## Supplementary Material

Supplementary Information

## Figures and Tables

**Table 1:** Heavy metal concentrations in water, sediments and soil for different land uses along the Shunde Waterway.

Land use and media	As	Hg	Cd	Zn	Pb	Cu	Cr	Ni
Soils	Mean ± Std. Deviation [Minimum, Maximum] (mg/kg)							
Riverbank vegetable plot (RVS)	72.3 ± 26.8	0.39 ± 0.12	2.25 ± 1.13	358.6 ± 249.6	100.3 ± 26.1	61.7 ± 20.4	71.5 ± 4.3	27.5 ± 5.2
	[34.7, 106.3]	[0.25, 0.59]	[1.02, 4.01]	[170.2, 907.7]	[59.0, 125.5]	[39.1, 99.6]	[66.7, 77.4]	[21.4, 36.2]
Riverbank green space (RGS) (0–20 cm)	42.5 ± 34.2	0.29 ± 0.10	1.02 ± 0.77	262.6 ± 354.9	80.6 ± 84.3	37.8 ± 21.6	66.8 ± 9.8	22.1 ± 5.0
	[14.9, 133.4]	[0.12, 0.45]	[0.28, 2.44]	[85.1, 1408.3]	[28.1, 324.7]	[10.4, 91.7]	[51.6, 87.4]	[14.6, 32.6]
Private vegetable plot (PVS) (0–20 cm)	17.5 ± 3.6	0.40 ± 0.41	0.55 ± 0.41	124.5 ± 59.3	36.8 ± 16.7	32.5 ± 17.8	68.6 ± 16.1	26.5 ± 10.8
	[12.6, 25.5]	[0.12, 1.43]	[0.12, 1.68]	[64.3, 289.1]	[21.1, 76.2]	[8.4, 66.3]	[42.1, 100.9]	[7.4, 42.8]
Public green space (PGS) (0–20 cm)	18.5 ± 7.6	0.24 ± 0.16	0.44 ± 0.22	111.6 ± 36.1	31.4 ± 12.0	18.8 ± 9.6	61.8 ± 10.8	19.2 ± 7.9
	[11.5, 47.0]	[0.03, 0.66]	[0.21, 1.16]	[59.1, 212.7]	[11.9, 56.0]	[5.1, 40.6]	[46.1, 78.7]	[3.0, 29.9]
Total Soils (0–100 cm)	27.8 ± 20.5	0.41 ± 0.24	1.29 ± 1.02	181.8 ± 158.0	52.5 ± 35.8	49.2 ± 28.1	73.3 ± 14.6	31.8 ± 7.3
	[11.6, 62.7]	[0.17, 0.74]	[0.43, 2.97]	[85.9, 501.5]	[26.9, 123.7]	[26.5, 103.9]	[52.0, 96.5]	[21.2, 41.0]
River								
surface sediments	47.8 ± 13.3	0.20 ± 0.12	1.76 ± 0.45	311.0 ± 118.1	93.3 ± 44.7	93.5 ± 58.4	106.0 ± 78.5	29.8 ± 8.0
	[22.7, 66.6]	[0.06, 0.51]	[0.65, 2.19]	[136.8, 481.8]	[29.8, 163.7]	[40.2, 236.0]	[54.2, 321.6]	[17.6, 39.8]
	Mean ± Std. Deviation [Minimum, Maximum] (μg/L)							
surface water	3.2 ± 1.1	0.02 ± 0.01	0.10 ± 0.07	12.6 ± 4.0	2.3 ± 2.5	3.8 ± 2.2	2.4 ± 4.3	2.3 ± 2.0
	[2.2, 5.4]	[0.01, 0.03]	[0.03, 0.25]	[5.9, 19.4]	[0.7, 9.0]	[2.0, 8.0]	[0.5, 14.6]	[1.2, 7.6]
Soil backgrounds	Heavy metal Background values in soil (mg/kg)^28^							
	16.2	0.26	0.44	109.6	50.1	43.8	62.0	32.6

**Table 2:** Hazard quotients of heavy metals in different media and land uses.

Land use and media	*HQ_ingestion_*			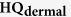			*HQ_inhalation_*			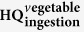			*THQ*		
	Mean	95th percentile	CV	Mean	95th percentile	CV	Mean	95th percentile	CV	Mean	95th percentile	CV	Mean	95th percentile	CV
RVS	0.207	0.392	47.5%	0.110	0.219	54.9%	0.024	0.038	35.8%	1.338	3.162	81.1%	1.678	3.567	66.3%
RGS	0.131	0.293	71.1%	0.046	0.114	81.6%	0.018	0.031	38.7%				0.195	0.408	62.2%
PVS	0.066	0.114	39.0%	0.032	0.059	45.5%	0.024	0.064	106.3%	0.476	1.061	67.6%	0.598	1.195	54.8%
PGS	0.065	0.119	45.7%	0.023	0.045	56.6%	0.015	0.033	68.3%				0.102	0.169	37.0%
Groundwater	0.570	1.457	94.8%										0.570	1.457	94.8%
Surface water	0.446	0.839	54.3%	0.001	0.002	83.0%							0.447	0.840	54.2%
River sediments	0.018	0.039	64.7%	0.053	0.143	87.7%							0.070	0.176	76.5%

**Table 3:** Contributions of heavy metal species to hazard quotients of the major exposure routes.

	As	Hg	Cd	Zn	Pb	Cu	Cr	Ni
RVS								
* HQ*_*ingestion*_	80.01%	0.43%	0.75%	0.39%	9.53%	0.51%	7.91%	0.46%
* * 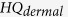	91.67%	0.09%	1.15%	0.03%	0.96%	0.03%	6.04%	0.03%
* HQ*_*inhalation*_	0.12%	98.63%	0.00%	0.00%	0.01%	0.00%	1.23%	0.00%
* * 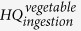	38.41%	0.30%	28.70%	4.81%	12.50%	4.11%	8.82%	2.35%
PVS								
* HQ*_*ingestion*_	60.68%	1.39%	0.57%	0.43%	10.98%	0.84%	23.73%	1.38%
* * 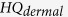	77.17%	0.34%	0.97%	0.04%	1.23%	0.05%	20.12%	0.09%
* HQ*_*inhalation*_	0.03%	98.77%	0.00%	0.05%	0.00%	0.00%	1.15%	0.00%
* * 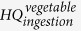	26.09%	0.86%	19.25%	4.69%	12.85%	6.08%	23.78%	6.40%
Groundwater								
* HQ*_*ingestion*_	84.49%	6.26%	2.30%	2.54%	0.22%	0.24%	0.00%	3.95%
Surface water								
* HQ*_*ingestion*_	84.86%	0.53%	0.80%	0.34%	5.31%	0.76%	6.50%	0.92%

**Table 4:** Sensitivity parameters for total hazard quotients of heavy metals in different media and land uses.

RANK	*THQ*_*RVS*_	*THQ*_*RGS*_	*THQ_PVS_*	*THQ_PGS_*	*THQ_Groundwater_*	*THQ_Water_*	THQ_Sediments_
1	*PUF*_*Cd*_ (0.475**)	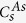 (0.864**)	*IR*_*v*_ (0.490**)	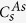 (0.653**)	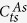 (0.647**)	*IR*_*w*_ (0.737**)	*EFsw* (0.683**)
2	*PUF*_*As*_ (0.429**)	*IR*_*s*_ (0.375**)	*PUF*_*As*_ (0.357**)	*IR*_*s*_ (0.610**)	*IR*_*w*_ (0.425**)	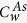 (0.548**)	*AFsd* (0.(0.551**)
3	*IR*_*v*_ (0.410**)	*AF*_*sa*_ (0.148**)	*PUF*_*Cr*_ (0.353**)	*AF*_*sa*_ (0.266**)	*DF* (0.352**)	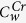 (0.259**)	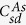 (0.267**)
4	*θ*_*v*_ (−0.257**)	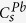 (0.141**)	*PUF*_*Cd*_ (0.323**)	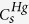 (0.260**)	*Kd*_*As*_ (−0.243**)	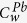 (0.132**)	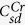 (0.102**)
5	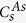 (0.256**)	BW (−0.078**)	*θ*_*v*_ (−0.315**)	BW (−0.144**)	BW (−0.055**)	BW (−0.131**)	*IR*_*sd*_ (0.084**)

^**^Correlation is significant at the 0.01 level (2-tailed).
