# Supplementary material for: Regional probabilistic risk assessment of heavy metals in different environmental media and land uses: An urbanization-affected drinking water supply area
Source: Sci Rep. 2016 Nov 15;6:37084. doi: 10.1038/srep37084 (PMC5109259; doi:10.1038/srep37084)
Supplement: Supplementary Information [file srep37084-s1.pdf]

Supporting information

Regional probabilistic risk assessment of heavy metals in different environmental media and land uses: An urbanization-affected drinking water supply area

Chi Peng[1], Yimin Cai[1], Tieyu Wang[1], Rongbo Xiao[2], Weiping Chen[1,] *

[1] State Key Laboratory of Urban and Regional Ecology, Research Center for Eco-environmental Sciences, Chinese Academy of Sciences, Beijing, 100085，People's Republic of China

[2] Guangdong Provincial Academy of Environmental Science, Guangdong 510045, China.

* corresponding author

Tel: +86-010-62843981

E-mail address: wpchen@rcees.ac.cn (*W.P. Chen*)

Table and figure list

Table S1 Mean, ranges and distribution of the exposure parameters.

Table S2 Mean and ranges of the contaminant-specific parameters.

Fig. S1 Distribution of sampling sites along the Shunde Waterway. (Map source: DigitalGlobe)

Table S1 Mean, ranges and distribution of the exposure parameters.

Variables	Symbol	Range	Distribution
Average body weight (kg)	BW	58.4 (43-77.1)	LN
Exposure duration (yr)	ED	30	Deterministic
Averaging time (d)	AT	30×365	Deterministic
Exposure Frequency for indoor activities (day/yr)	EF_{ia}	350	Deterministic
Exposure Frequency for outdoor activities (day/yr)	EF_{oa}	225	Deterministic
Exposure Frequency for swimming (day/yr)	EF_{sd}	18 (0-79)	Triangular
Exposure time for swimming (hr/24 hr)	ET_{sd}	2/24	Deterministic
Leaf vegetable water content	θ_v	0.936 (0.905-0.956)	Triangular
Water-filled soil porosity (Lwater/Lsoil)	θ_w	0.25 (0.05-0.5)	LN
Dry soil bulk density (g/cm3)	ρ_b	1.5 (1.4-1.6)	Triangular
Dilution factor	DF	0.08 (0.022-0.27)	LN
Soil Ingestion Rate (mg/day)	IR_s	65 (1-200)	LN
Sediment Ingestion Rate (mg/day)	IR_{sd}	50 (1-100)	LN
Total Water Ingestion Rate (mL/day)	IR_w	1957 (788-3650)	Triangular
leaf vegetable ingestion rate (mg/day)	IR_v	295 (110-416) × 10^3	Triangular
Air inhalation rate (m3/day)	IR_a	15.5 (11.6-19.7)	Triangular
Soil to skin adherence facotor for farmers (mg/cm2)	AF_{sf}	0.1 (0.01-0.4)	LN
Soil to skin adherence facotor for adults (mg/cm2)	AF_{sa}	0.07 (0.01-0.3)	LN
Sediment to skin adherence facotor (mg/cm2)	AF_{sd}	0.16 (0.01-1)	LN
Skin surface area available for exposure in outdoor Activities (m2)	SA_{oa}	0.495 (0.473-0.594)	Triangular
Skin surface area available for exposure in swimming (m2)	SA_{sd}	1.5 (1.3-1.8)	Triangular
Particulate Emission Factor (m^3/kg)	PEF	1.36×10^{-9}	Deterministic

Table S2 Mean and ranges of the contaminant-specific parameters.

	Hg	As	Cd	Zn	Pb	Cu	Cr	Ni
PUF (unitless)	0.01 (0.0005-0.0716)	0.007 (0.00015-0.103)	0.535 ± 0.667	0.176±0.191	0.019±0.01	0.116±0.054	0.016±0.017	0.074±0.058
K_d (L/kg)	150 (52-200)	606 (125-1780)	400 (75-4300)	130 (62-530)	46622 ± 27808	2805 ± 948	3.7×10^{6} (1.8×10^{6} - 4.3×10^{6})	250 (65-1900)
RfD_{ing}	3.0×10^{-4}	3.0×10^{-4}	1.0×10^{-3}	3.0×10^{-3}	3.5×10^{-3}	4.0×10^{-3}	3.0×10^{-3}	2.0×10^{-2}
RfD_{inhal}	8.6×10^{-5}						2.9×10^{-5}	
RfD_{dermal}	2.1×10^{-5}	1.2×10^{-4}	1.0×10^{-5}	6.0×10^{-3}	5.3×10^{-4}	1.2×10^{-3}	6.0×10^{-5}	5.4×10^{-3}
K_p	1.00×10^{-3}	1.00×10^{-3}	1.00×10^{-3}	6.00×10^{-4}	1.00×10^{-3}	1.00×10^{-3}	1.00×10^{-3}	2.00×10^{-4}
ABS	0.03	0.001	0.001	0.001	0.001	0.001	0.001	0.001

PUF (unitless): Plant uptake factor, RfD (mg kg^{-1} day^{-1}): Chronic oral reference dose, K_d (L/kg): Soil-water partition coefficient, K_p (cm/hr): Dermal permeability constant, ABS (unitless): Dermal absorption factor

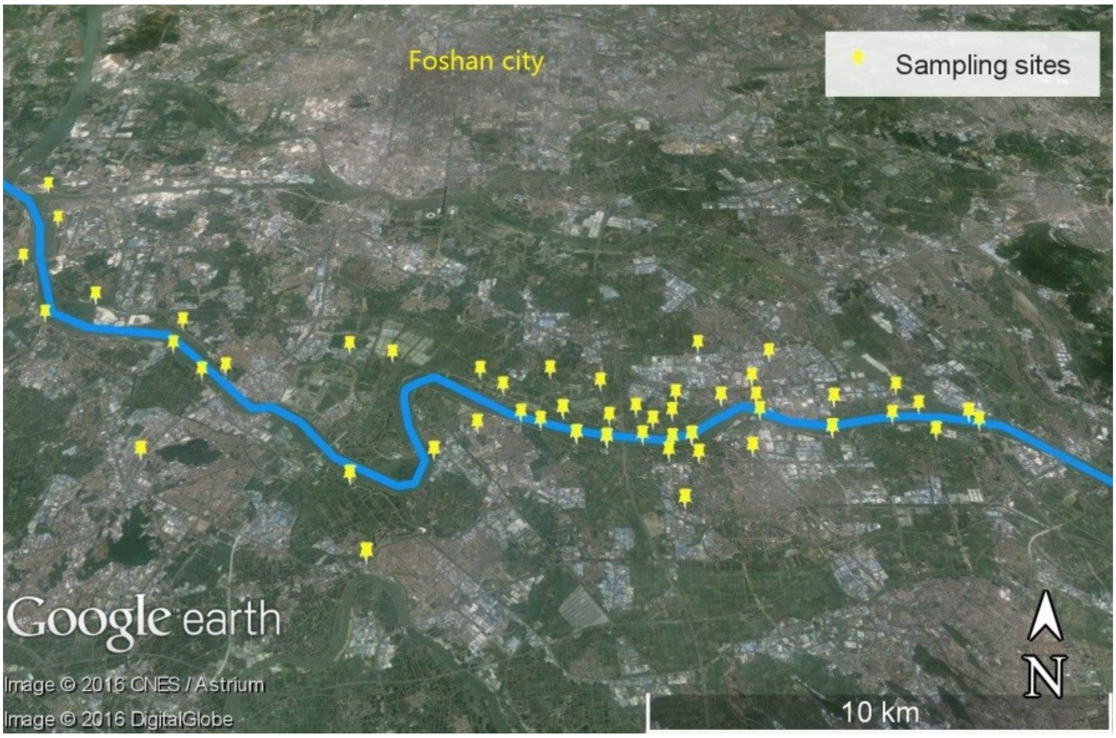

Fig. S1 Distribution of sampling sites along the Shunde Waterway. (Map source: DigitalGlobe)
